# Atlanta Academy of Medicine

**Published:** 1879-02

**Authors:** H. F. Scott

**Affiliations:** Reporter


### Reports of Societies.

### ATLANTA ACADEMY OF MEDICINE.

H. F. SCOTT, M.D., REPORTER.

ATLANTA, GA., January 13, 1879.

Academy met, President-elect, Dr. J. S. Todd, in the chair. On taking the chair, Dr. Todd made an instructive and interesting address to the members, thanking them for the honor conferred in electing him to preside over their body, and exhorting them to more regularity in attendance in the future.

Dr. Calhoun said he wished to exhibit to the members a rare specimen of disease. The patient, a lady, had received, some eight or ten years previous, a blow on the left cheek, fracturing molar and nasal bones of that side. Since that time there had been a more or less constant swelling and inflamation, together with a discharge of pus. Some two years ago this discharge became very copious.

He was consulted some seven weeks ago, and on examination found the eye-ball protruding from its socket and hanging down on the cheek; there was ecchymosis of upper lid and severe pain, which had been relieved by morphine-eating. From long exposure of the cornea to the air the eye had been rendered useless and was accordingly enucleated.

The original trouble has thereby not been relieved, no progress whatever ensuing, either for the better or worse, except the relief of the pain; there has been a sloughing of the entire lower lid and nasal bones.

He does not consider the case as of syphilitic origin; it has every appearance of a malignant nature, although he cannot state positively that it is so.

In conclusion, he desired the members to carefully examine the case and express their opinion as to its nature.

Dr. Bak was impressed with the slowness of its action, and is inclined to believe it a slow necrosis following the contusions of the blow; its late occurrence after receipt of the injury could be explained by the fact that flat bones necrose and suppurate slower than the large ones; was of the opinion that when all of the bruised bone had been thrown off the case would progress favorably.

The President remarked that the discussion of this case brought forward the important question as to whether traumatism alone be capable of producing cancer.

Dr. Bak said many cases of *carcinoma mammale* were conceded to have been the result of blows.

Dr. Baird thought that traumatism of a chronic nature, *i. e.* such as maintain a morbid condition for some time in contradistinction to acute injuries, which quickly disappear, might eventuate in the deposit of cancer cells.

Dr. Alexander said this was virtually admitting that blows are capable of originating cancer.

The President does not understand Dr. Baird to mean that all chronic injuries were, or would be, followed by cancerous changes—for instance, that a fracture of the tibia would have such a termination.

Dr. Baird illustrated his position by cases in which cancer of the penis had been caused by the irritation of a lengthy prepuce and accumulation of products of Tyson's glands.

Dr. Calhoun's experience leads him to agree with Dr. Baird. He remembers several cases in point—one where the continuous application of caustics to a pterygium resulted in cancer of the eye-ball.

Dr. Low's experience also leads him to concur in the views of Dr. Baird. Cancer was often extremely difficult to diagnose, and medical practitioners were often at variance as to the nature of a particular case. Instanced a case in his own experience where there had been the widest diversity of opinion amongst physicians of repute.

Dr. J. W. Griggs cannot speak from experience, but thinks the constitution of the patient should be taken into consideration—for instance, where there is a cachexia, the depraved blood repeatedly coming in contact with the source of irritation, develops cancer.

Dr. Park's opinion is that the first case of cancer must have originated from some trauma, and had then become engrafted on the constitution and been handed down to succeeding generations.

Dr. J. T. Johnson thinks a great many cases are not of a hereditary nature, but that irritation of any kind may eventuate in cancer.

Dr. Rohe hasn't any doubt cancer can be of traumatic origin. The structure of epithelioma differed from epithelium only in the different arrangement of its component cells, and therefore any injury to the epithelium may cause that arrangement of its cells characteristic of cancer. The only instance recorded of primary cancer of bone is that by Stricker, in his recent work on pathology. *Its* cancerous nature, however, has been contended against by some, who are of the opinion that it was merely a bone-cyst.

Dr. Salm thinks it can be of a traumatic origin; related a case confirmatory of this view occurring in Dr. Paul Eve's clinic, where an injury to bones of arm degenerated into a cancer.

Dr. Bak quoted Rokitansky as an authority that chronic irritation *could* produce cancer.

The President is not prepared to disagree with the opinions expressed by the gentlemen, still he thinks there are weighty arguments in favor of all cases being hereditary. It might be very difficult to determine in many cases, but if examined into, thought evidences of its existence in patient's ancestry could be secured.

Dr. J. T. Johnson propounded the following question to the President, to-wit: A person receives a blow which was followed by cancer. Said person's grandfather had likewise been afflicted with cancer. Now, do you not admit the former case might have been of local origin and independent of any hereditary taint?

Dr. Alexander wished to know if all cases are hereditay, why wasn't the disease developed in every member of the family.

The President believes persons inherit from one or both of their parents a constitutional predisposition to cancer and other diseases. But, just as in consumption, the offspring may inherit the peculiarities of either their father or mother, one of whom may have been entirely free from hereditary taint. In such an instance its failing to show itself in certain members of the family, and its exhibiting itself in others, would be satisfactorily accounted for.

Dr. Rohe said, morphologically, lupus was nearest allied to cancer, and related a case where lupus was present in mother and daughter; still lupus was regarded by the best authorities as not being hereditary.

Dr. Bak said a cancer, if extirpated, will return, while lupus, if removed, does not recur. Said sarcoma was nearer cancer, pathologically, than lupus. He admits that trauma might be its cause, but not in every case.

The President said whilst this subject was under discussion he would ask if arsenic, in the opinion of the members, has influence on the development of cancer. Stated that Dr. J. T. Johnson and himself had recently had a case where they recommended extirpation, but the patient was induced, after consultation by a respectable practitioner, to try arsenic.

Dr. J. T. Johnson said, as is well known, years ago arsenic was much relied upon, and even at the present day some writers believe it beneficial; it certainly is decidedly tonic.

Dr. Bak thought certain remedies had the power of relieving suffering and invigorating patient, and therefore should be used even if they are unable to cure. Stated that Dr. Friedreick, of Berlin, in a case of *carcondina ventriculi* used condurago with great benefit, succeeding in ameliorating the distress.

Dr. Rohe believes arsenic used internally of great benfit in the first stages of cancer. Applied it locally in a case where nitrate of silver, etc., has been repeatedly applied; thought he had thoroughly destroyed it, still it was as palpable as ever. Gave 10 to 12 drops Fowler's Solution *ter die*, and caused it to retrograde.

Dr. J. T. Johnson thought if anywise beneficial it should be used.

Dr. Calhoun said, except as a tonic arsenic had no more influence over cancer than so much quinine.

The President thought if one recovered on arsenic, there had been a mistaken diagnosis.

Dr. Baird has from time to time mentioned cases illustrative of the value of galvanism in various diseases. He simply desired to record the fact that he had recently secured relief in a number of cases of facial neuralgia and sciatica. About these cases nothing of special interest existed, but the results of treatment are of practical importance, and should be known generally by the profession.

Dr. Griggs inquired if Dr. Baird had ever used it in spasmodic stricture of the urethra, stating it had been recommended by Dr. Otis.

The President asked Dr. Baird if neuralgic parts were generally sensitive to pressure; thought they were relieved by pressure.

Dr. Baird replied that a broad, firm pressure relieved neuralgia, but pressure with the points of the finger is painful; relies on galvanism alone in many cases, whilst in others use it in connection with other remedies.

The President stated he had recently found tr. guiac to be very beneficial in tonsillitis, and recommended it to members for trial. Given in half-drachm doses every four or five hours.

Dr. J. T. Johnson said it was recommended in Brown's

"Diseases of the Throat" for various affections, used both internally and locally.

Academy adjourned.

---

ATLANTA, GA., January 20, 1879.

Dr. P. R. Cortelyou reported the following case of epilepsy, the result of a fracture of os frontis, nineteen years previous, cured by long-continued use of sulphate of atropine:

In November, 1873, I was called to attend F. W. Moores, aged 32 years, during an attack of typhoid fever, and while visiting him was informed by his wife, who had been married to him about two years, that during that time he had been subject to epileptic convulsions, which had been increasing in frequency till, before the attack of fever, they occurred several times a week, and that his mind was becoming considerably affected by them. He had been under the care of several physicians, and Dr. Brown-Sequard among them. He had prescribed the bromides in large doses with strychnine, and applied small blisters to nape of neck and head. Thus far no treatment had the slightest effect on the disease. During the continuance of the typhoid fever the fits did not occur, but as soon as convalescence was established they returned.

Upon tracing very carefully the history of the patient, I learned that in 1860, while working in the navy yard machine shop at Boston, endeavoring to replace a band on a drum, he was thrown from a platform to the ground, a distance of 17 feet, striking his head on the left side against the corner of an iron box, thus causing a fracture of the os frontis about one inch in length, with depression, being badly comminuted and the eye protruding from the socket. Severe convulsions immediately set in, lasting several hours. After a day or two he complained of severe pain in left ear, which was followed by very offensive straw colored discharge. He also had a good deal of hemorrhage from posterior nares. At this time, no one could walk across the room or touch the bed without the patient complaining of intense pain. There was also great intolerance of light. All the physicians who saw him at the time were of the opinion that there was also a fracture of the skull, and considered his case hopeless. These facts were taken from the Boston *Medical Journal*, in which an account of the accident was published.

Upon questioning some members of his family, I learned that at times he had been subject to fainting spells more or less frequently, and on account of them was obliged to leave the naval service, in which he was employed. His family had concealed his trouble, so that his wife was not aware of his former history, and therefore thought the epilepsy of recent occurrence. On examination, I found a depression of bone on left side of temple about an inch and a half in length. The question of trephining was considered, and upon consultation with Dr. James R. Wood, of New York, it was deemed unadvisable, on account of the extent and location of depression. Therefore, as he had tried all other methods of treatment without benefit, I recommended a trial of sulphate of atropine in gradually increasing doses, according to the plan of Prof. Trouseau, of Paris. I informed his wife that it would require great patience and long treatment, but it might succeed, and was worth a trial as a last resort. She consented, and agreed to carry out faithfully all instructions, January, 1874. I then ordered the following:

℞.—Atropine sulph., . . . . . gr i.
Spts. vin. gallici, . . . . . m 100.

M. S.—One drop at dose once a day—in the morning, as the fits occurred chiefly in the day time. This was increased by one drop at the beginning of every month until he took 1-10 of a grain daily. The first effect of the medicine was to quiet excessive muscular twitching at night, thus giving patient calmer and more beneficial rest. Next the fits, instead of occurring two or three times a week, came only once. Then an interval of ten days elapsed. They also were not severe. The interval between them continued to increase with an occasional relapse to old condition. After he had been using medicine for about one year, fits did not take place oftener than once in three months. When patient had taken 1-10 grain atropine daily for a month throat and eye symptoms appeared, showing that the drug had been carried to maximum dose. It was then gradualy reduced. After having been under treatment for seventeen months fits seemed to have been reduced. The medicine and patient moved to the country, where he employed most of his time in garden work.

Since October, 1875, he has had no recurrence of fits; his mental condition has also improved so that it is nearly as good as it ever was, so his friends say. The case is interesting, from the fact that all other methods of treatment had been tried, without success. Again, on account of long standing of disease and the cause of its commencement. How did the belladonna act? It seems to me by quieting nervous irritability, and thus breaking up the habit of the nervous system to giving way to irritation; in fact, altering the nerve force, rendering it less susceptible to irritation.

Prof. Trouseau does not consider the treatment as a specific by any means, but says in his hands it has proved the least inefficacious of any he has tried, though often failing. Another point he makes is: "That the remedy is to be trusted in so far, as it is to be administered in accordance with certain rules, which should not be infringed on. When a disease has deeply permeated the organism, one cannot cure it in a short space of time." It is necessary for success to have a careful and competent person to carry out directions to the letter. My patient's wife did not fail a single time in the seventeen months in giving the medicine, and at the prescribed time. Is not then this method of treatment worthy of further trial, especially when all other means have failed?

P. R. CORTELYOU, M.D.

*Atlanta, Ga.*

